# Author Correction: Medical history predicts phenome-wide disease onset and enables the rapid response to emerging health threats

**DOI:** 10.1038/s41467-025-56429-1

**Published:** 2025-02-10

**Authors:** Jakob Steinfeldt, Benjamin Wild, Thore Buergel, Maik Pietzner, Julius Upmeier zu Belzen, Andre Vauvelle, Stefan Hegselmann, Spiros Denaxas, Harry Hemingway, Claudia Langenberg, Ulf Landmesser, John Deanfield, Roland Eils

**Affiliations:** 1 Department of Cardiology, Angiology and Intensive Care Medicine, Deutsches Herzzentrum der Charité (DHZC), Berlin, Germany; 2 Charité – Universitätsmedizin Berlin, corporate member of Freie Universität Berlin and Humboldt Universität zu Berlin, Klinik/Centrum, Berlin, Germany; 3 Computational Medicine, Berlin Institute of Health (BIH), Charite - University Medicine Berlin, Berlin, Germany; 4 Friede Springer Cardiovascular Prevention Center@Charite, Charite - University Medicine Berlin, Berlin, Germany; 5 Institute of Cardiovascular Sciences, University College London, London, UK; 6 Center for Digital Health, Berlin Institute of Health (BIH), Charite - University Medicine Berlin, Berlin, Germany; 7 MRC Epidemiology Unit, Institute of Metabolic Science, University of Cambridge, Cambridge, UK; 8 Precision Health University Research Institute, Queen Mary University of London and Barts NHS Trust, London, UK; 9 Institute of Health Informatics, University College London, London, UK; 10 Institute for Medical Engineering and Science, Massachusetts Institute of Technology, Massachusetts, USA; 11 Pattern Recognition and Image Analysis Lab, University of Münster, Münster, Germany; 12 British Heart Foundation Data Science Centre, London, UK; 13 Health Data Research UK, London, UK; 14 National Institute for Health Research, Biomedical Research Centre at University College London Hospitals, London, UK; 15 Berlin Institute of Health (BIH), Charite - University Medicine Berlin, Berlin, Germany; 16 DZHK (German Centre for Cardiovascular Research), Partner Site Berlin, Berlin, Berlin, Germany; 17 Health Data Science Unit, Heidelberg University Hospital and BioQuant, Heidelberg, Germany

**Keywords:** Disease prevention, Statistics, Epidemiology

Correction to: *Nature Communications* 10.1038/s41467-025-55879-x, published online 10 January 2025

The authors wish to clarify that this article^1^ is a revised version of a retracted article^2^ and has undergone peer review.
