# Supplementary material for: Medical history predicts phenome-wide disease onset and enables the rapid response to emerging health threats
Source: Nat Commun. 2025 Jan 10;16:585. doi: 10.1038/s41467-025-55879-x (PMC11724087; doi:10.1038/s41467-025-55879-x)
Supplement: Supplementary file 1 — Supplementary Information [file 41467_2025_55879_MOESM1_ESM.pdf]

Supplementary Figure 1: Characterisation of routine health records

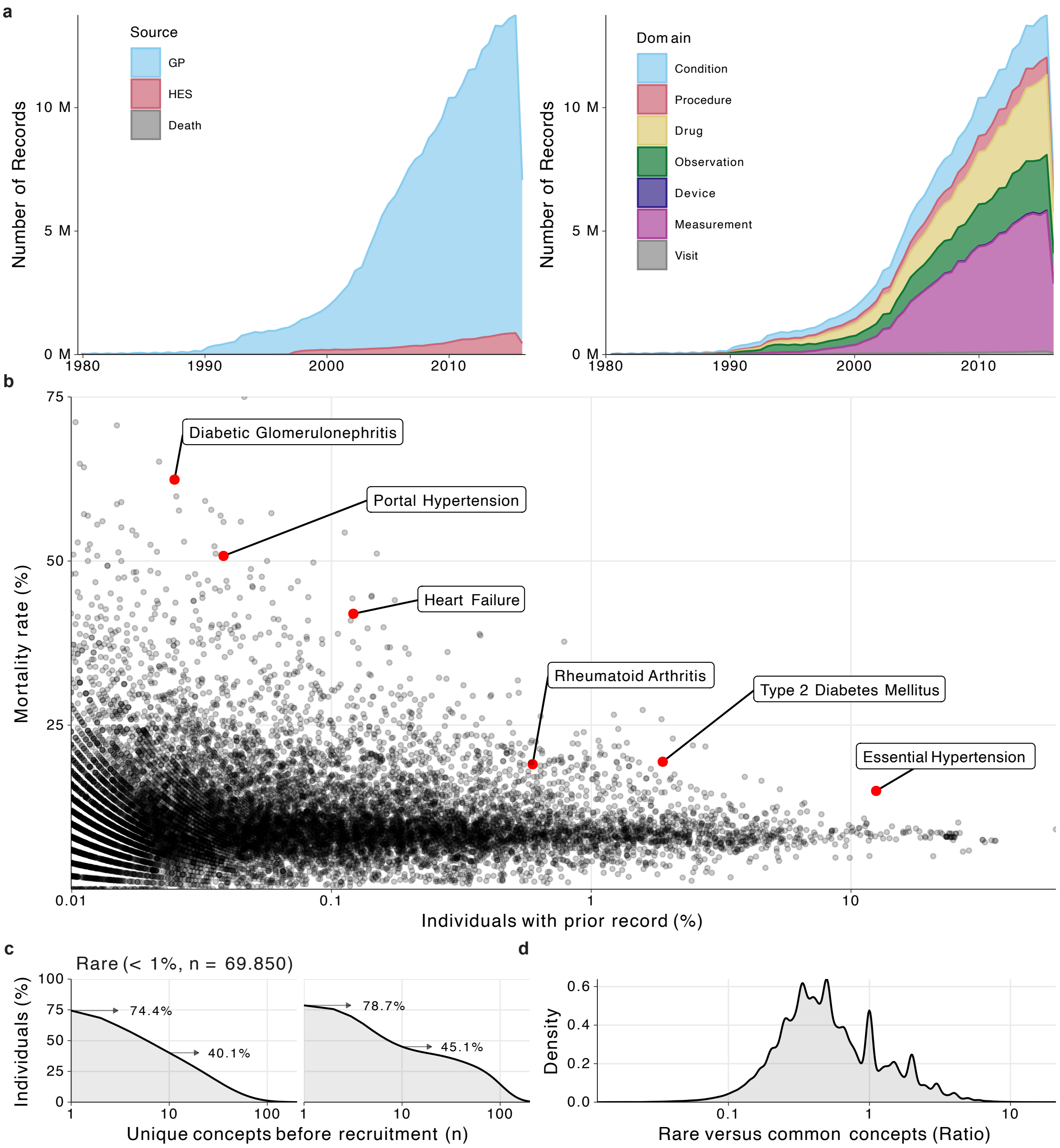

a) Yearly counts of health records stratified by GP (red), hospital (blue), and death records (yellow). **c)** Yearly counts of health records stratified by record domain. **c)** Mortality rate conditional on prior records. Highlighted are high-risk records with gradually increasing frequency. **d)** Percentage of individuals with prior rare or common records. **e)** Ratio of rare and common records per individual. Statistical measures were derived from 502,489 individuals. Source data are provided as Source Data file.

Supplementary Figure 2: Comparison of our approach with the linear baseline

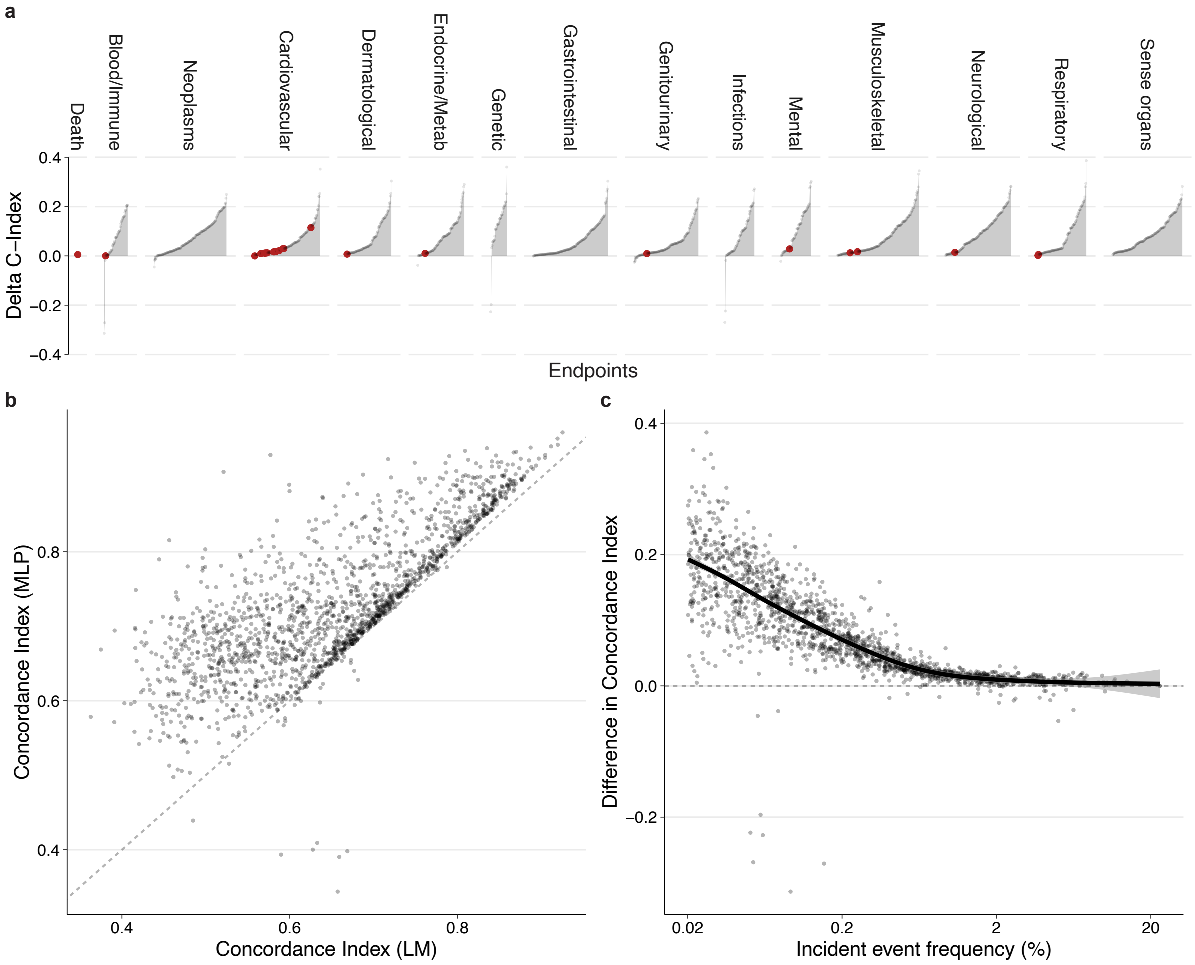

a) Differences in discriminatory performance quantified by the C-Index between the linear baseline risk model (LM) and our approach with a multi-layer perceptron (MLP) for all 1,741 endpoints. We found significant improvements for 1,530 (87.9%), nonsignificant differences for 59 (3.4%), and significantly worse results for 20 (1.1%) of the 1,741 investigated endpoints. **b)** Direct comparison of the absolute C-Index for the linear model (LM, x-axis) and our approach (MLP, y-axis). **c)** Improvement of our approach (y-axis) by the incident event frequency of the endpoint. The improvements over the linear model are largest for rarer and smallest for common endpoints. Statistical measures were derived from 502,489 individuals. Source data are provided as Source Data file.

Supplementary Figure 3: Comparison with established comorbidities and risk factors

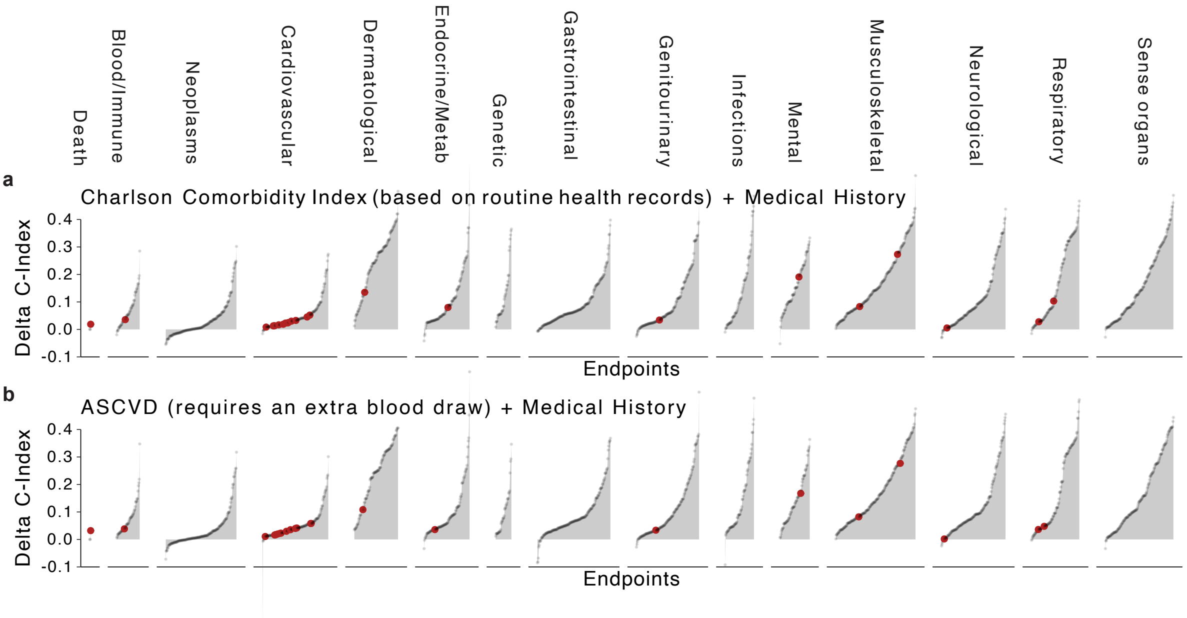

a) Differences in discriminatory performance quantified by the C-Index between CPH models trained on Age+Sex+Comorbidities from the Charlson Comorbidity Index (CCI) compared with the added medical history for all 1,741 endpoints. We found significant improvements for 1,516 (87.1%) of the 1,741 investigated endpoints. **a)** Differences between CPH models trained on ASCVD risk factors only compared with the added medical history for all 1,883 endpoints. We found significant improvements for 1,459 (83.8%) of the 1,741 investigated endpoints. Red dots indicate the 24 highlighted endpoints. Statistical measures were derived from 502,489 individuals. Source data are provided as Source Data file.

Supplementary Figure 4: Individual predicted phenome-wide risk profiles

a

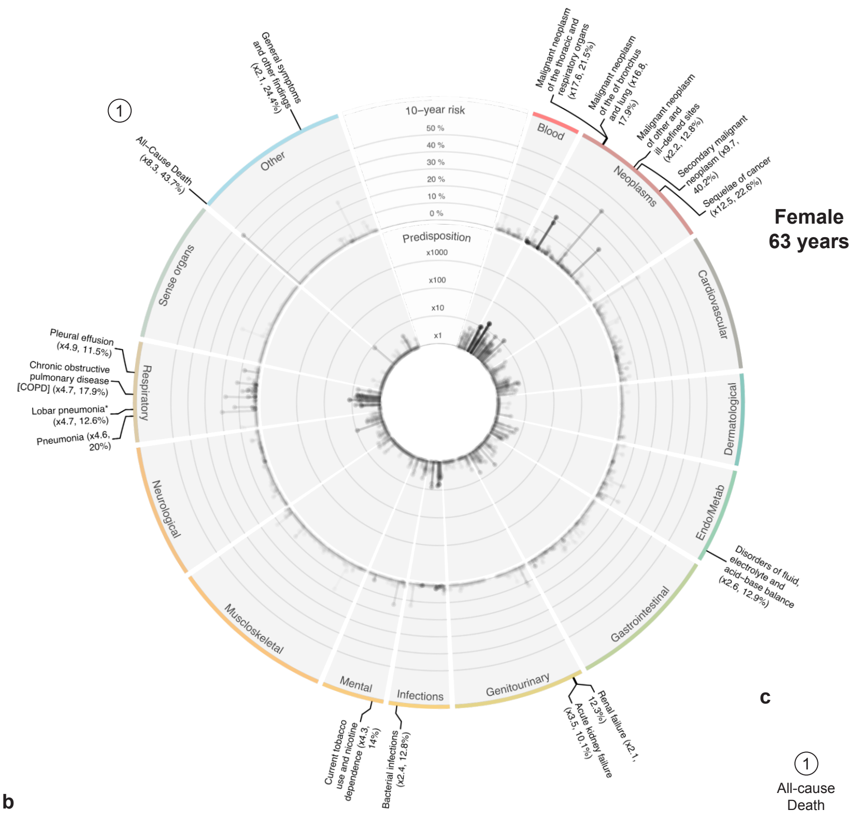

b

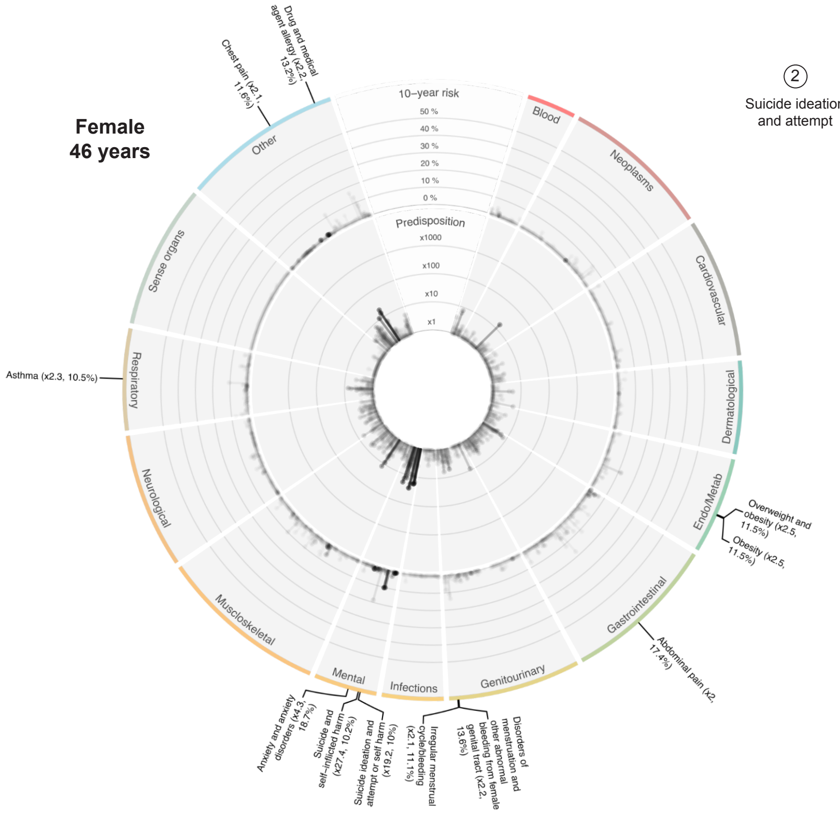

c

Highest attributed records over the study population:

(1) All-cause Death	Multiple sclerosis<br>Cancer care review<br>Seen in hematology clinic<br>Intermittent claudication<br>Non-Hodgkin's lymphoma
(2) Suicide ideation and attempt	Bipolar disorder<br>Mental health review<br>Mental health medication review<br>Drug overdose<br>On severe mental illness register

a+b) Example of individual predicted phenome-wide risk profile for a 63-year-old (a) and a 46-year-old female (b). Predisposition (10-year risk estimated by Age+Sex+RiskState compared to risk estimated by Age+Sex alone) is displayed in the inner circle, and absolute 10-year risk estimated by Age+Sex+RiskState can be found in the outer circle. Labels indicate endpoints with a high individual predisposition (> 2 times higher than the Age+Sex-based reference estimate) and absolute 10-year risk > 10%. e) Top 5 highest attributed records for selected endpoints.

Supplementary Figure 5: Individual predicted phenome-wide risk profiles

a

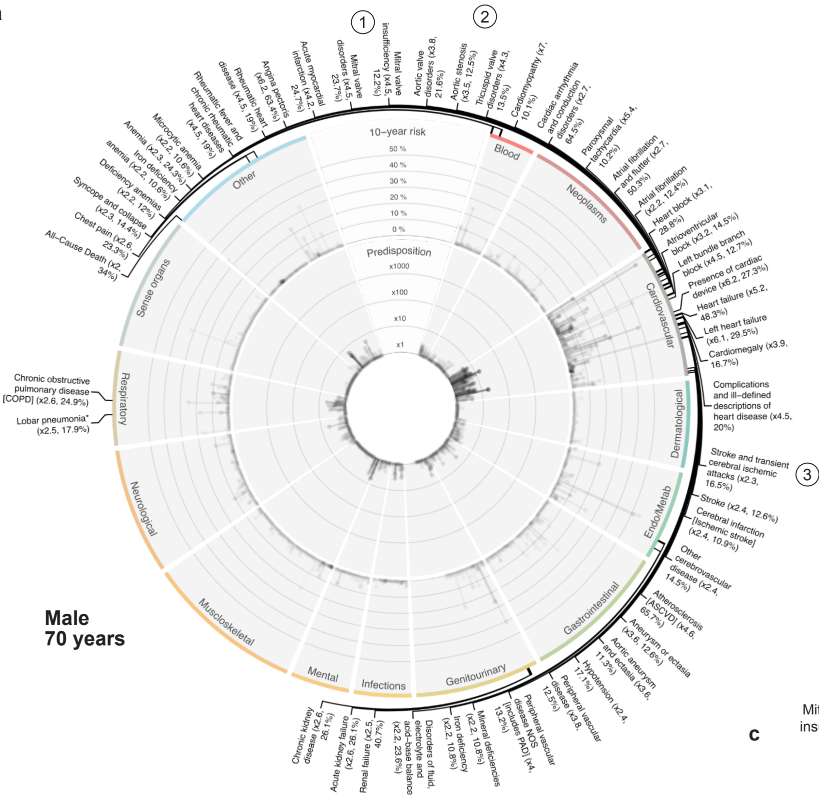

b

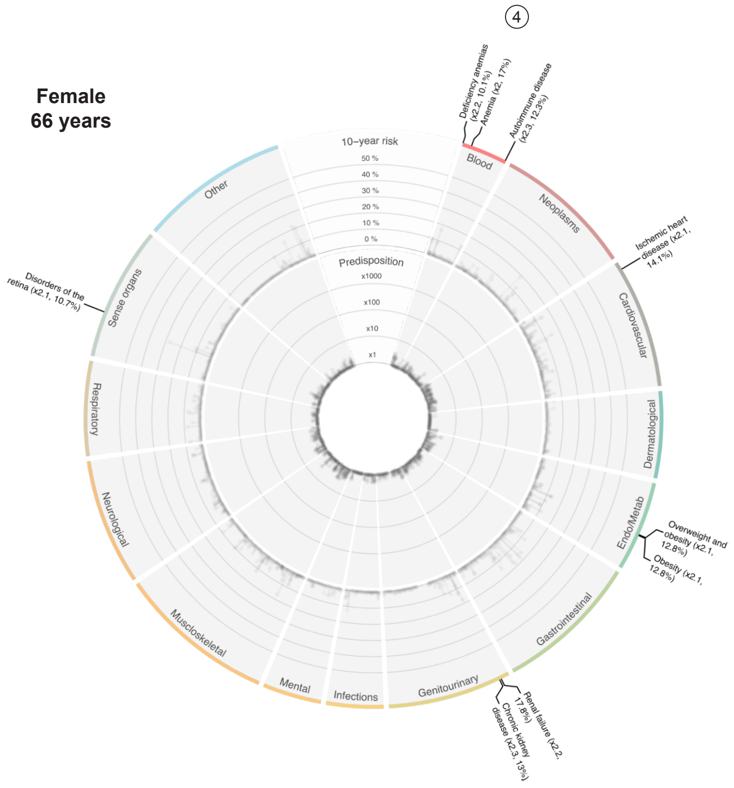

c

Highest attributed records over the study population:

① Mitral valve insufficiency
- Echocardiogram abnormal
- Atrial arrhythmia
- Systolic Murmur
- Echocardiography
- Referral to cardiology service

② Aortic stenosis
- Echocardiogram abnormal
- Intermittent claudication
- Chronic kidney disease stage 3
- Atrial arrhythmia
- Renal failure syndrome

③ Stroke
- Epilepsy
- Intermittent claudication
- Hemiplegia
- Epilepsy medication review
- Pneumococcal vaccination invitation letter sent

④ Anemia
- Seen in hematology clinic
- Crohn's disease
- Diagnostic extraction of bone marrow
- Multiple sclerosis
- Type 2 diabetes mellitus without complication

a+b) Example of individual predicted phenome-wide risk profile for a 70-year-old male (a) and a 66-year-old female (b). Predisposition (10-year risk estimated by Age+Sex+RiskState compared to risk estimated by Age+Sex alone) is displayed in the inner circle, and absolute 10-year risk estimated by Age+Sex+RiskState can be found in the outer circle. Labels indicate endpoints with a high individual predisposition (> 2 times higher than the Age+Sex-based reference estimate) and absolute 10-year risk > 10%. e) Top 5 highest attributed records for selected endpoints.

Supplementary Figure 6: Stability of model performance with the removal of recent information

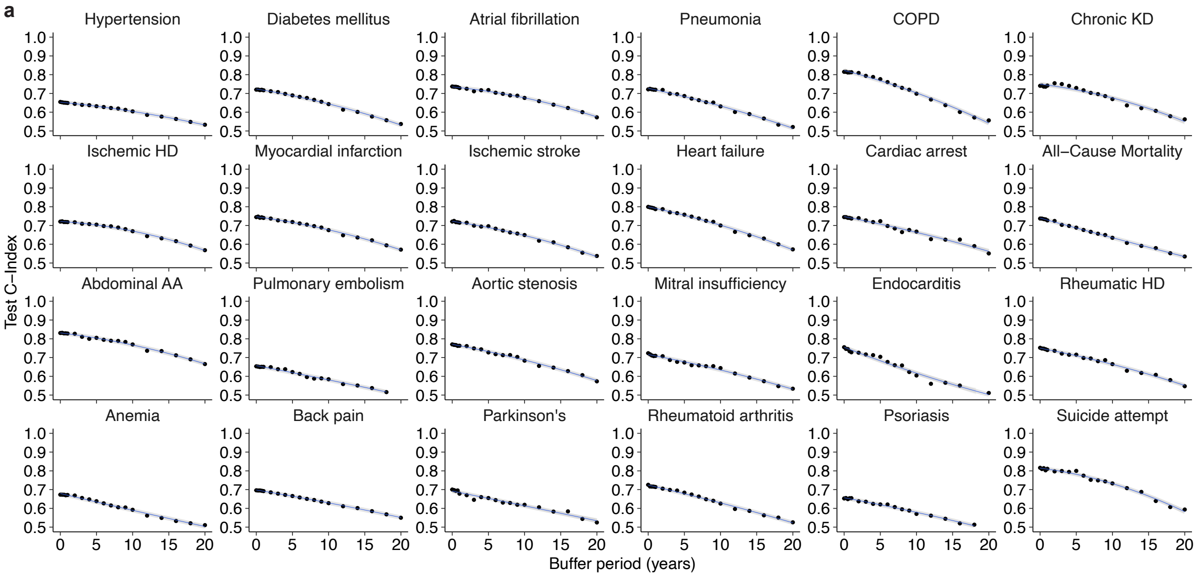

Discrimination performance of the medical history, measured in terms of absolute C-Indices, subjected to progressive data removal deprivation. For each time point, a model is trained and evaluated using a dataset from which records documented within a specified time frame prior to recruitment - denoted on the 'Buffer Period (Years)' axis - have been removed. The performance gradually declines in performance across all endpoints, indicating that the model effectively incorporates both the individuals' long-term medical history and recent interactions with the healthcare system in order to predict future disease onset. Statistical measures were derived from 502,489 individuals. Source data are provided as Source Data file.
