# Supplementary material for: Medical history predicts phenome-wide disease onset and enables the rapid response to emerging health threats
Source: Nat Commun. 2025 Jan 10;16:585. doi: 10.1038/s41467-025-55879-x (PMC11724087; doi:10.1038/s41467-025-55879-x)
Supplement: Supplementary file 2 — Description of Additional Supplementary Files [file 41467_2025_55879_MOESM2_ESM.pdf]

Description of Additional Supplementary Files

Supplementary Data 1: Endpoints in this study

List of endpoints in this study with associated metadata.

Supplementary Data 2: Medical History Predictors in this study

List of medical history predictors in this study with associated metadata.

Supplementary Data 3: Reference Predictors in this study

List of clinical reference predictors in this study with metadata.

Supplementary Data 4: Incident Event Stratification

Incident events stratified by risk deciles across all study endpoints.

Supplementary Data 5: Discriminative Performance of the Medical History Scores

Discriminative Performance (quantified by Harell's C-Index) of scores based on medical history beyond reference scores, including Age+Sex, ASCVD, and Age+Sex+Clinical Comorbidities. Harell's C-Index was calculated with the lifelines package by bootstrapping the aggregated test sets within ten years after recruitment. Differences are considered significant when multiple-testing Bonferroni-adjusted confidence intervals do not overlap.

Supplementary Data 6: Hazard Ratios of the Medical History Scores

Hazard Ratios of scores based on medical history beyond reference scores including Age+Sex, ASCVD, and Age+Sex+Clinical Comorbidities. Adjusted and unadjusted hazard ratios

were calculated by bootstrapping the Cox proportional hazard model results with the lifelines package.

Supplementary Data 7: Discriminative Performance of the Medical History Scores to compared to established scores for the Primary Prevention of Cardiovascular Disease

Discriminative Performance (measured by Harell's C-Index) of the Medical History scores beyond the established cardiovascular risk scores SCORE2, ASCVD, and QRISK3 for a selection of cardiovascular disease endpoints. Harell's C-Index was calculated with the lifelines package by bootstrapping the aggregated test sets within ten years after recruitment. Differences are considered significant when multiple-testing Bonferroni adjusted confidence intervals do not overlap.

Supplementary Data 8: Discriminative performance in the All of Us cohort

Discriminative Performance (measured by Harell's C-Index) of the Medical history-based scores after transferring the unmodified risk models to the All of Us cohort. Harell's C-Index was calculated with the lifelines package by bootstrapping the aggregated test sets within ten years after recruitment. Differences are considered significant when multiple-testing Bonferroni-adjusted confidence intervals do not overlap.

Supplementary Data 9: Feature attributions for 24 selected endpoints

Feature attributions are estimated by approximating Shapley values for 24 selected endpoints. Shapley values are averaged (so-called local attributions to quantify importance for affected individuals) and summed (global attributions to quantify importance for population ranking). Higher Shapley values indicate higher importance in the model.
